# Stroke-like Symptoms With a Cardiac Clue

**DOI:** 10.1016/j.acepjo.2025.100315

**Published:** 2026-01-10

**Authors:** Emma Alley, Mark Olaf

**Affiliations:** Department of Emergency Medicine, Geisinger Medical Center, Danville, Pennsylvania, USA

**Keywords:** stroke, electrocardiogram, cerebral, T waves

## 1 Case Presentation

**Figure 1:**
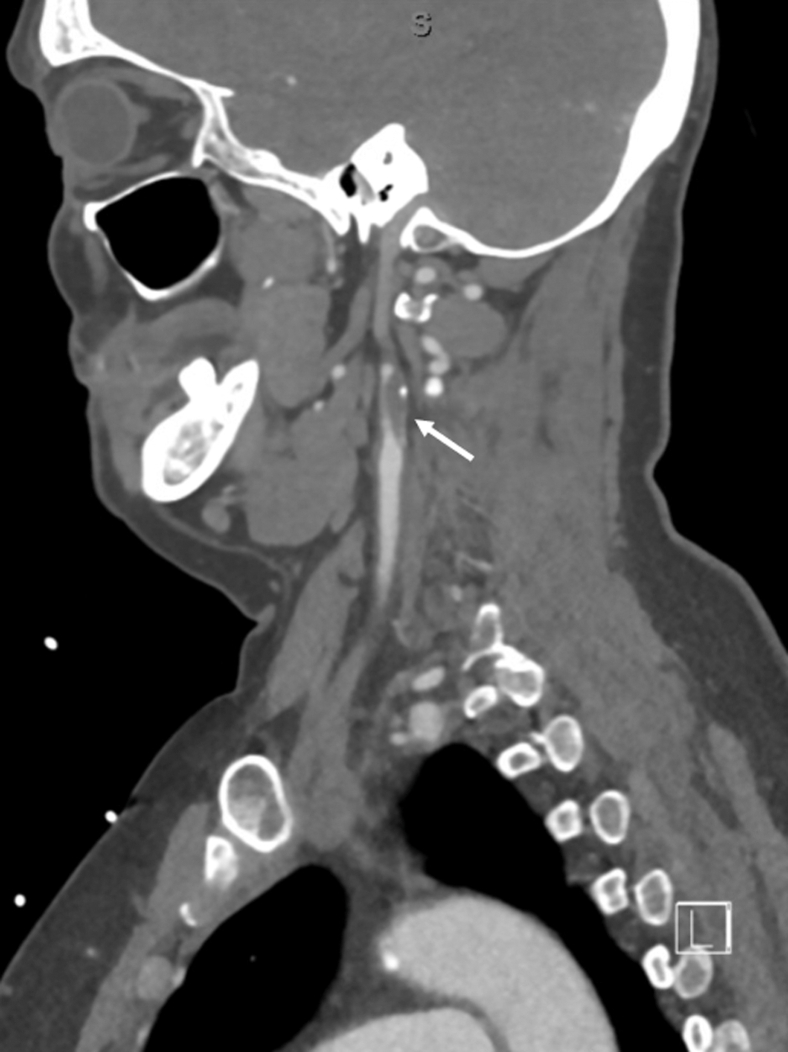
Sagittal view of a CT angiography of the head and neck. The arrow points to the left internal carotid artery in an area with 95% stenosis. The patient is noted to have both a hard and soft plaque in the area of stenosis. CT, computed tomography.

**Figure 2:**
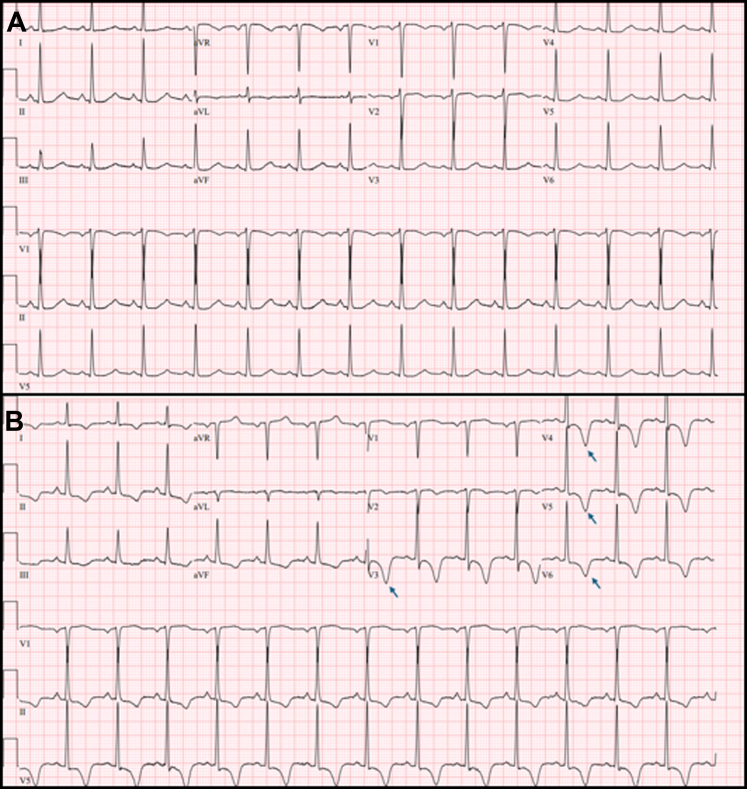
Depiction of 2 EKGs for the patient. (A) Baseline EKG from 3 years prior to presentation. (B) Taken at the time of presentation for right arm weakness; shows diffuse T-wave inversions that are most pronounced in the lateral leads. The deep T-wave inversions in the lateral leads are labeled with arrows. EKG, electrocardiogram.

A 57-year-old woman presented to the emergency department for evaluation of headache and right arm weakness that began 6 hours prior to arrival. She had a past medical history of hypertension and a transient ischemic attack 13 years prior. Her blood pressure was noted to be 190/111. Neurologic examination demonstrated right arm weakness with ataxia, decreased sensation, and lower extremity drift. A stroke alert was initiated, and computed tomographic angiography (CTA) of the head was obtained with findings seen in Figure 1. An electrocardiogram (EKG) was obtained as part of the stroke evaluation and was compared with a prior EKG as seen in Figure 2.

### 1.1 Diagnosis: 95% Left Internal Carotid Stenosis With Cerebral T Waves on EKG

The patient was diagnosed with 95% left internal carotid stenosis causing critical stenosis with stroke symptoms. The EKG findings were consistent with cerebral T waves. Cerebral T waves are a rare finding in cases of increased intracranial pressure, hemorrhagic stroke, and ischemic stroke. A study by Stone et al^1^ found that approximately 2.1% of individuals presenting with stroke had cerebral T waves. In some reports, cerebral T waves were present on EKG up to 12 hours before patients developed focal neurologic deficits.^2^ Although cerebral T waves have been well documented in the literature, the mechanism of how intracranial insult can cause cardiac dysfunction is not well understood. In patients with diffuse T-wave inversions, differentials should include acute intracranial abnormalities, pulmonary embolism, diffuse myocardial ischemia, ventricular hypertrophy, and bundle branch blocks.^3^

## Funding and Support

By *JACEP Open* policy, all authors are required to disclose any and all commercial, financial, and other relationships in any way related to the subject of this article as per ICMJE conflict of interest guidelines (see www.icmje.org). The authors have stated that no such relationships exist.

## Conflict of Interests

All authors have affirmed they have no conflicts of interest to declare.
